# Species-specific phylloplane responses to changes in external pH

**DOI:** 10.1093/jxb/eraf157

**Published:** 2025-04-14

**Authors:** Cristal López-González, Jean-Baptiste Floc’h, Tanya Renner, Kadeem J Gilbert

**Affiliations:** Department of Plant Biology, W. K. Kellogg Biological Station, Program in Ecology, Evolution, & Behavior, and Plant Resilience Institute, Michigan State University, Hickory Corners, MI, USA; Department of Plant Biology, W. K. Kellogg Biological Station, Program in Ecology, Evolution, & Behavior, and Plant Resilience Institute, Michigan State University, Hickory Corners, MI, USA; Research Centre for Ecological Change, University of Helsinki, Helsinki, Finland; Department of Entomology, The Pennsylvania State University, University Park, PA, USA; Department of Plant Biology, W. K. Kellogg Biological Station, Program in Ecology, Evolution, & Behavior, and Plant Resilience Institute, Michigan State University, Hickory Corners, MI, USA; Department of Entomology, The Pennsylvania State University, University Park, PA, USA; The James Hutton Institute, UK

**Keywords:** Abiotic stress, *Beta vulgaris*, buffering ability, comparative transcriptomics, *Gossypium*, leaf pH, *Nepenthes*, pH stress, phylloplane

## Abstract

The leaf surface, known as the phylloplane, represents the initial point of contact for plants in their interactions with the above-ground environment. Although previous research has assessed how leaves respond to variations in external pH, particularly in the context of acid rain, there remains a limited understanding of the molecular mechanisms through which plants detect, respond to, and mitigate cellular damage. To examine plant responses to changes in external pH, we selected five species known to have a range of phylloplane pH values from alkaline to acidic under normal conditions, and investigated the response of the phylloplane pH to treatments at pH 6.5, 4, and 2. We found that plants were able to modify their phylloplane pH, and that this buffering ability was species-specific; however, only *Gossypium* species displayed strong buffering. When leaves were exposed to either pH 6.5 or pH 4, the *Gossypium* species alkalinized the phylloplane pH to slightly higher values than the dry control pH but, remarkably, when leaves were exposed to pH 2, they buffered the pH to 6 within 5 min. Transcriptional analysis further indicated that the responses to external pH changes varied among the five species, with differentially expressed genes associated with Ca^2+^-signaling pathways as well as Ca^2+^- and H^+^-ATPases pumps being highlighted. These findings also suggested that pH stress affected photosynthesis, and that both wetness and moderate pH shifts might trigger additional abiotic and biotic stress-signaling pathways.

## Teaser

Comparative transcriptomics analysis across species with a broad range of leaf-surface pH indicates the molecular mechanisms underlying differing buffering capacities in response to external pH changes.

## Introduction

The outermost surface of a leaf, known as the phylloplane, is the initial point of interface for plants with the above-ground external environment (Shepherd and Wagner, 2007; Gilbert and Renner, 2021). Plants must face a multitude of external abiotic environmental factors on these surfaces, such as temperature and moisture (Pereira, 2016; Kashyap *et al.*, 2021; Zhang *et al.*, 2022, 2023; Munns and Millar, 2023). How physiological changes in evapotranspiration alter leaf temperature has been well studied, including the ecological consequences of the resulting heterogeneity in the leaf microenvironment (Xue *et al.*, 2020; Kibler *et al.*, 2023; Griffani *et al.*, 2024). Another factor of likely importance is pH, which influences the chemical reactions that occur in multiple systems (Tsai and Schmidt, 2021). Leaves can regularly experience external changes in pH, either through precipitation or other fluids that come into contact with the leaf in nature, and it should therefore be advantageous for plants to be able to sense external pH changes and respond appropriately to counter harmful changes, such as their impact on photosynthetic activity and their potential to cause cellular damage (Cornelissen *et al.*, 2006, 2011; Tao *et al.*, 2019; Rodríguez-Sánchez *et al.*, 2020; Tsai and Schmidt, 2021). The ability of plants to control pH has mainly been studied in the rhizosphere (Husson, 2013; Schmidt, 2022), where soil pH can affect the availability of mineral nutrients for absorption, the structure of the soil microbiome, and other plant abiotic and biotic interactions (Husson, 2013; Lammel *et al.*, 2018; Muneer *et al.*, 2021; Tsai and Schmidt, 2021; Schmidt, 2022). Given its relevance to the rhizosphere, the regulation of external pH should also be an important subject of study in the phylloplane, due to its frontline role in sensing and interacting with environmental changes above ground. Previous studies have shown that the phylloplane pH can play a role in how plants mitigate the effects of acid rain, fertilizers and pesticides sprayed onto the leaf, and potential plant–microbe interactions (Simon *et al.*, 1994; Wong, 1997; Shepherd and Wagner, 2007; Lager *et al.*, 2010; Tao *et al.*, 2019; Shi *et al.*, 2021; Perreault and Laforest-Lapointe, 2022). Furthermore, the phylloplane pH can vary between lineages and species, and is influenced by moisture (Cornelissen *et al.*, 2011; Gilbert and Renner, 2021). Most plants tend to have a neutral pH, for example *Beta vulgaris*. Only species belonging to *Malvaceae* have been reported to have a highly alkaline phylloplane pH under normal conditions (Harr *et al.*, 1980; Gilbert and Renner, 2021), the levels increase further after spraying water on the leaf surface (Harr *et al.*, 1980; Navon *et al.*, 1988; Smith *et al.*, 1996). The opposite extreme of phylloplane pH is observed in carnivorous plants, ranging from just below pH 5 to an extreme of pH 1 (Gilbert *et al.*, 2020; Gilbert and Renner, 2021).

Simulated acid rain has been the most common type of experiment related to the disturbance of phylloplane pH, and these studies have shown that leaves can neutralize acidic pH and mitigate foliar damage (Adams and Hutchinson, 1984; Knittel and Pell, 1991; Liang *et al.*, 2015; Ren *et al.*, 2018; Rodríguez-Sánchez *et al.*, 2020; Shi *et al.*, 2021). This buffering ability is species-specific, and its effectiveness depends on the acidity and duration of the external disturbance (Adams and Hutchinson, 1984; Pfanz and Heber, 1986; Liu *et al.*, 2013; Liang *et al.*, 2015; Zheng *et al.*, 2017; Ren *et al.*, 2018). The ability to actively regulate phylloplane pH might have consequences for how the leaf surface interacts with biotic and abiotic stressors, such as in microbial interactions (Perreault and Laforest-Lapointe, 2022; Schmidt, 2022), and thus it can play a role in the fitness and productivity of agricultural crops (Bashir *et al.*, 2022). Despite this, the molecular mechanisms behind the plant response to pH as a stressor have not been studied in detail. Fundamental questions remain unanswered, including whether there are any specific pathways involved in the response, whether the mechanism converges with other abiotic stresses, and whether the primary mechanism is shared across plant taxa.

Here, we use a comparative transcriptomic approach to elucidate the key genes involved in pH-buffering ability in order to identify fundamental, generalizable principles regarding phylloplane functioning. This requires looking broadly across ‘physiologically typical’ species (e.g. *Beta*) as well as more extreme models (e.g. *Gossypium* and *Nepenthes*) in a search for insights into the limits that a plant can potentially withstand. We selected a panel of five species with known variation in predicted dry phylloplane pH as reviewed by Gilbert and Renner (2021), ranging from alkaline to acidic extremes (*Gossypium arboreum*, *G. hirsutum*, *Beta vulgaris*, *Nepenthes bicalcarata,* and *N. rafflesiana*). Due to the rapid ability of plants to buffer pH, as shown by Harr and Guggenheim (1995), we tested the response to acidic pH disturbance in the leaf blade over a period of 5 min. Studies examining rapid responses to stress are needed to understand how plants manage to adapt to a changing environment, but there are only a few reports of fast transcriptional reprogramming in response to an external stimulus such as wounding or heat stress (Hander *et al.*, 2019; Lohani *et al.*, 2022). Given that phylloplane pH regulation has been observed in diverse plant species (Gilbert and Renner, 2021), our expectation was that the molecular response to pH treatments is based on differential gene expression in pathways that are common to all plants, as opposed to unique genes. We anticipated that examining differential gene expression relative to the control would reveal changes in pathways that include plasma membrane H^+^-ATPases (Müller *et al.*, 1996; Fuglsang *et al.*, 2007; Liang *et al.*, 2015; Cosse and Seidel, 2021; Seidel, 2022) and ABA (Van Volkenburgh and Davies, 1983), together with other pathways associated with protecting the leaf tissue from pH stress-induced damage (Lager *et al.*, 2010; Ren *et al.*, 2018). On the other hand, unexpected pathways could be involved that reveal different and species-specific mechanisms of pH alteration. As a result of our experiments, we found that the response to pH disturbance is interlinked with the general signaling pathway involved in other abiotic stresses and that the response to pH disturbance is species-specific.

## Materials and methods

### Plant material and treatments

We acquired seeds of *Gossypium arboreum* (accession PI 615701), *G. hirsutum* (accession PI 529181), and *Beta vulgaris* (accession Ames 3060) from the USDA Germplasm Resources Information Network (GRIN; https://www.ars-grin.gov/). The seeds were planted in Sunshine Mix #4 Professional Growing Mix, (Sun Gro Horticulture, Agawam, MA, USA) and grown in a greenhouse at The Pennsylvania State University under the following conditions: 16/ 8 h day/night cycle at 24/21 °C, under ambient lighting plus 100 mmol m^–2^ s^–1^ PAR supplemental illumination. Because it was not feasible to grow *Nepenthes* from seed, we attained small adult plants (clonally propagated) of *N. bicalcarata* (accession BE-3031) and *N. rafflesiana* (accession BE-3722) from Borneo Exotics ltd (supplied by Carnivero, Austin, TX, USA). The *Nepenthes* plants were potted in a sphagnum medium and grown in a Conviron PGR15 reach-in growth chamber under the following conditions: 12/ 12 h day/night cycle at 28/26 °C, relative humidity 80%, fluorescent/incandescent illumination at 250 mmol m^–2^ s^–1^. The growing medium was treated with imidacloprid to clear a scale infestation before the start of the experiment. The species grown from seed were all sown in October 2020 and grown until and the plants had produced the first two nodes of true leaves (4–6 weeks after sowing). We aimed to perform all the pH manipulation experiments on the same day on the youngest fully expanded leaves to minimize temporal/ontogenetic variation.

For the pH manipulation experiments, the leaf was sprayed with one of three pH treatments: pH 6.5, 4.0, or 2.0. The sprays were created by mixing distilled deionized water (ddH_2_O) with the appropriate volume of HCl to reach the target pH, and the solutions were stored in inert glass spray bottles to avoid leaching of material or changes in pH due to CO_2_ diffusion. The leaf was sprayed until thoroughly wet (~15 sprays) and the pH response of the leaf was recorded after the 5 min exposure period using a flat-tipped pH probe (HI981037; Hanna Instruments Inc., Leighton Buzzard, UK). The leaves were then immediately collected directly into 2 ml DNAse/RNAse-free tubes of RNALater^®^ (ThermoFisher Scientific) after 5 min of exposure. Autoclaved forceps wiped with RNAse *AWAY*™ (Invitrogen) were used to pull the leaf from the abscission zone of the petiole, or in the case of *Nepenthes* lamina from the weak point of the stem (pitchers were removed 4–5 d prior to the experiment). For each species, we sampled three replicate plants per treatment, including a dry (unsprayed) control. The samples stabilized in RNALater® were stored at 4 °C until subsequent extraction.

### RNA extraction and sequencing

We conducted total RNA extractions using a Spectrum™ Plant Total RNA Kit (Sigma-Aldrich), according to manufacturer’s protocol with some modifications. First, the leaves were transferred into clean tubes and centrifuged at 7197 *g* for 30 s to remove the remaining RNALater®. For the mechanical lysis step, we used a single large steel bead (autoclaved and sterilized with RNAse *AWAY™*) to homogenize the leaf sample in Lysis Solution (Sigma-Aldrich) using a MP Biomedicals FastPrep-24™ machine set to 5.5 m s^–1^ for 20 s. Following homogenization, the sample was incubated in a heat block at 56 °C for 5 min, and the remaining steps were carried out according to the RNA Kit protocol. The extracted RNA was sent to Novogene for library preparation, quality control, and sequencing on the Ilumina NovaSeq 6000 PE150 platform.

### Transcriptomic analyses

RNA-seq analyses were performed for each species separately. The raw read quality of each paired-end library was tested using FastQC v0.11.9 (http://www.bioinformatics.babraham.ac.uk/projects/fastqc/; Andrews, 2010). Adapters and low-quality bases were removed using Trimmomatic (Bolger *et al.*, 2014). Transcriptome *de novo* assembly was performed using Trinity v2.9.1 (Grabherr *et al.*, 2011) with default settings for each species to treat them the same. Read counts were done with Salmon v1.5.0 (Patro *et al*., 2017). Low counts (<3TPM) were filtered out and the most highly expressed isoform for each gene was kept. TransDecoder (Haas *et al.*, 2013) was used to identify the ORF coding regions in all the assemblies. Annotation was done with Trinotate v4.0.2 (Haas *et al.*, 2013) and EnTAP v1.0.1 (Hart *et al.*, 2020), mainly using the Swiss-Prot and PFAM protein databases. The Salmon counts were filtered by taxonomy, with only genes that were annotated for Viridiplantae taxa being considered.

### Differential gene expression analysis

Differential gene expression (DGE) analysis was performed for each species separately using DESeq2 v1.38.3 (Love *et al*., 2014; Zhang *et al.*, 2018; Emms and Kelly, 2019). Batch correction for each species was performed with COMBAT_Seq v0.0.4 (Zhang *et al.*, 2018).

In addition to the unsprayed dry control (Dry), the pH 6.5 sample was considered as the wet control (Wet) due to its pH being close to that of distilled water. DGE analysis was performed to compare each pH treatment (Wet, pH 4, pH 2) against the dry control (WetvsDry, pH4vsDry, pH2vsDry) and to compare the pH 4 and pH 2 treatments against the wet control (pH4vsWet, pH2vsWet). Differentially expressed genes (DEGs) for each species were extracted with a cut-off of |log_2_(fold-change)| >1 and an adjusted *P*-value of <0.05. Graphic representation of the number of DEGs was done using the ComplexUpset package v1.3.3 in RStudio v4.2.3 and visualization of metabolic pathways was done with MapMan v3.5.1 (Thimm *et al.*, 2004). Gene ontology (GO) enrichment analysis was conducted using ClueGo v2.5.9 (Bindea *et al.*, 2009) via Cytoscape v3.10.2 (Otasek *et al.*, 2019). As *G. hirsutum* was the only species from our study to have an available GO-term database in ClueGO, we identified orthologs for each of the DEGs in the other species. Enriched pathways for each group of DEGs compared with the Dry control and with the Wet control were obtained for each species and visualized with Cytoscape.

### Orthologous groups across species and cluster analysis

Protein alignments from the TransDecoder protein output for each species were used together with the protein sequences from the three reference genomes available (*G. arboreum*, *G. hirsutum*, and *B. vulgaris*) to find orthologous matches across species using OrthoFinder v2.5.4 (Emms and Kelly, 2019). The protein sequences of the reference genomes were downloaded from EnsemblPlants for *B. vulgaris* (v1.2.2; https://plants.ensembl.org/) and from NCBI (https://ftp.ncbi.nlm.nih.gov/genomes/all/; both accessed 20 February, 2023) for *G. hirsutum* (v2.1; accession GCF_007990345.1) and *G. arboreum* (v2; accession GCF_025698485.1). The orthogroup IDs file together with the gene expression dataset were used to perform a co-expression clustering analysis with Clust v1.18.0 (Abu-Jamous and Kelly, 2018) to identify co-expressed gene clusters across species.

### Statistical analysis

Statistical analyses were performed in RStudio v4.2.3, and consisted of one-way ANOVA followed by Dunnett’s or Fisher’s LSD tests, and generalized linear modeling.

## Results

### Ability of plants to regulate phylloplane pH

**Fig. 1.**
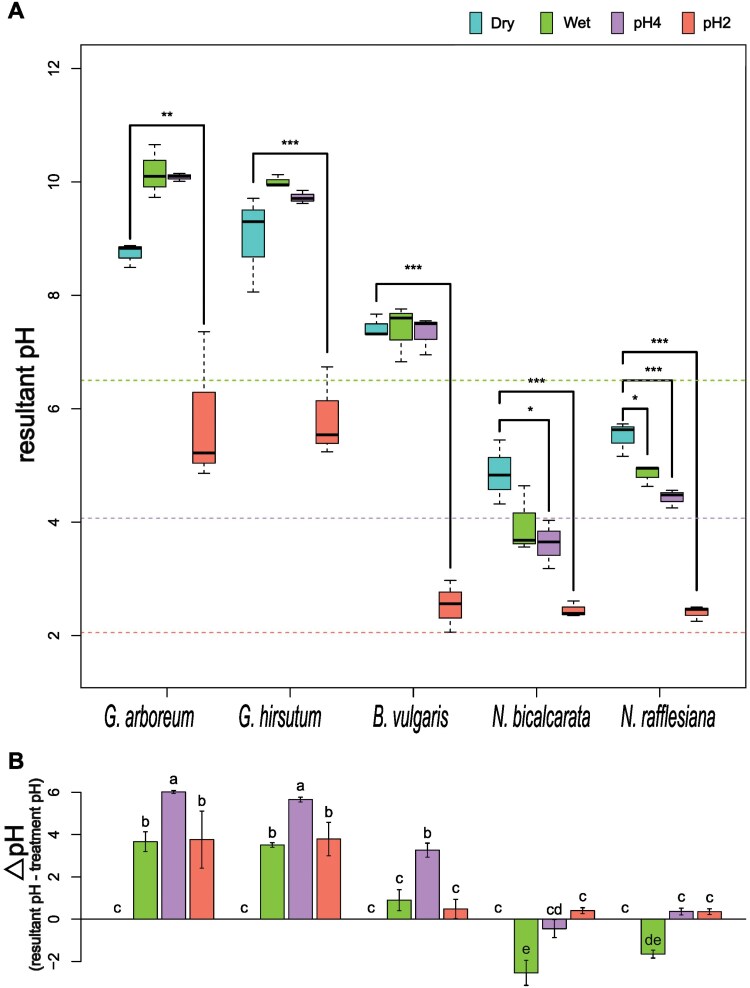
Buffering responses of the phylloplane to changes in external pH differ among the species. The pH of the leaf surface of *Gossypium arboreum*, *G. hirsutum*, *Beta vulgaris*, *Nepenthes bicalcarata*, and *N. rafflesiana* was determined (Dry control), and then they were sprayed with HCl solutions of pH 6.5 (Wet control), pH 4, and pH 2, and the resultant pH of the phylloplane was determined after 5 min. (A) Boxplots of the resultant pH. Significant differences compared with the Dry control were determined using one-way ANOVA followed by Dunnett’s test: *P<0.05, **P<0.01, ***P<0.001; *n*=3. (B) The buffering responses of the species, quantified as the difference between the resultant pH and the treatment pH. Positive values indicate an alkalinizing response, negative values indicate an acidifying response. Different letters indicate significant differences between treatments within each species, as determined using one-way ANOVA followed by Fisher’s LSD test (*P*<0.05).

The five different species were selected for their range of normal phylloplane pH levels as reviewed by Gilbert and Renner (2021). Both of the *Gossypium* species had an initial (dry, unsprayed) phylloplane pH that was alkaline (*G. arboreum* 8.7±0.1; *G. hirsutum* 9.2±0.4), while *Beta vulgaris* was nearly neutral (7.4±0.1) and both *Nepenthes* species were acidic (*N. bicalcarata* 4.8±0.3; *N. rafflesiana* 5.5±0.6; Fig. 1A). The nearly neutral pH of *B. vulgaris* is similar to most other plants (Gilbert and Renner, 2021), and hence it can be considered as the ‘physiologically typical species’ in our experiment against which the other species can be compared (Supplementary Table S1). We expected the responses to the Wet (pH 6.5, neutral control) and pH4 treatments to differ due to the increase in acidity, but interestingly within each species there were no significant differences (Fig. 1A). This showed that all of the species were able to buffer their pH effectively under the pH4 treatment. *Nepenthes bicalcarata* acidified its phylloplane in response to both Wet (3.96±0.6) and pH4 (3.62±0.4) treatments. Although the resultant pH levels did not differ between the Wet (*P*=0.59) and pH4 (*P*=0.65) treatments within species, they did vary across species/genera (<0.001; Supplementary Table S1).

Certain pH response patterns were observed at the genus level. Although the effect was not significant, both the *Gossypium* species increased pH slightly compared with the dry control under both Wet and pH4 treatments (Fig. 1A). *Beta vulgaris* buffered both the Wet and pH4 treatments to similar values relative to the dry control. Finally, both the *Nepenthes* species showed the opposite response to *Gossypium*, in that they acidified their pH under the Wet and pH4 treatments, by as much as 2 units for *Nepenthes*. The pH2 treatment significantly affected the resultant pH for all the species (Fig. 1A; Supplementary Table S1); however, the *Gossypium* species were able to significantly buffer the effect by ~4 units of pH (Fig. 1B). The buffering responses varied across the species and were pH-dependent.

### Differential gene expression

To identify key genes involved in the response to external pH changes, we performed RNA-seq analysis. After filtering transcripts with low counts (<3 TPM) and selecting only the most expressed transcript per gene, we ended up with 96 097 transcripts (13.064%) for *G. arboreum*, 64 429 (16.93%) for *G. hirsutum*, 53 788 (12.35%) for *B. vulgaris*, 85 027 (14%) for *N. bicalcarata*, and 124 547 (14.48%) for *N. rafflesiana*. We annotated genes using EnTAP and selected only those annotated as Viridiplantae. The number of genes remaining were 16 742 for *G. arboreum*, 17 239 for *G. hirsutum*, 11 274 for *B. vulgaris*, 12 719 for *N. bicalcarata*, and 13 325 for *N. rafflesiana*.

**Fig. 2.**
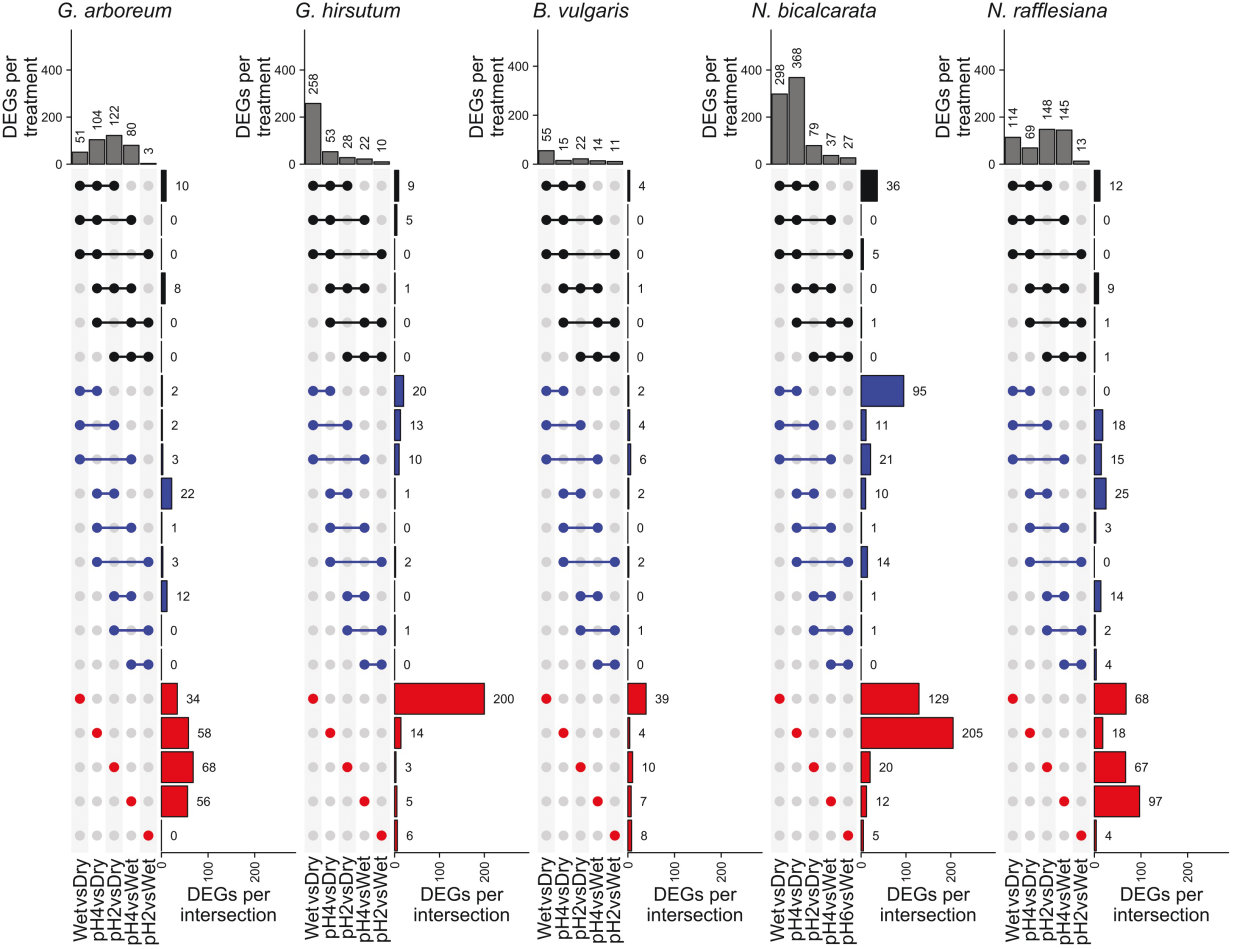
The number of differentially expressed genes (DEGs) in leaves in response to changes in external pH varies between the species and the different pH treatments. The pH of the leaf surface of *Gossypium arboreum*, *G. hirsutum*, *Beta vulgaris*, *Nepenthes bicalcarata*, and *N. rafflesiana* was determined (Dry control), and then they were sprayed with HCl solutions of pH 6.5 (Wet control), pH 4, and pH 2, and the resultant pH of the phylloplane was determined after 5 min. The total numbers of DEGs in the comparisons for each species are summarized at the top of the figure, and the numbers of shared (intersecting) DEGS between the different comparisons are shown below. Black indicates three-way comparisons; blue indicates pairwise comparisons; and red indicates DEGs that were unique to individual comparisons. DEGs were selected with a cut-off of |log_2_(fold-change)|>1 and adjusted *P*-value of <0.05.

We conducted differential gene expression analysis with DESeq2 using a multifactorial array consisting of 1) comparing each pH treatment against the dry control in order to identify genes involved in the general disturbance to wetness and pH, and 2) comparing the pH4 and pH2 treatments against the Wet (pH 6.5) treatment to identify genes that could be more specifically related to pH changes (Fig. 2). We identified a total of 279 DEGs for *G. arboreum*, 290 for *G. hirsutum*, 90 for *B. vulgaris*, 567 for *N. bicalcarata*, and 358 for *N. rafflesiana* across all treatment comparisons, with a cut-off parameter of log_2_(FC)≥|1| and false discovery rate of <0.05. Each species showed different numbers of DEGs across treatment comparisons, with *B. vulgaris* having the fewest DEGs both in general and across treatments; WetvsDry (55 DEGs) was the one comparison that contained more than half the total found in this species. Interestingly, *N. bicalcarata* showed the highest number of DEGs of all the species, and the pH4vsDry comparison (368 DEGs) seemed to drive more than half of them.

Most DEGs were uniquely expressed in only one-treatment comparisons, with fewer genes shared between the two- or three-treatment comparisons (Fig. 2). Genes involved in a general response to external disturbance and wetness were expected to be amongst those shared in all three of the WetvsDry, pH4vsDry, and pH2vsDry comparisons, while genes specifically related to pH responses were expected to be amongst those shared in the pH4vsDry, pH2vsDry, pH4vsWet, and pH2vsWet comparisons, and to be uniquely differentially expressed in the pH2vsWet comparison. However, no clear pattern was observed. Both species of *Gossypium* had a similar number of total DEGs, but their distribution among the treatment comparisons was quite different, with the most extreme difference being observed in *G. hirsutum* in the pH6vsDry comparison, where there was a total of 258 DEGs, most of which (200) were uniquely expressed.

### Analysis of orthologues

We used OrthoFinder (Emms and Kelly, 2019) to determine which genes were orthologues across species. We included the reference genomes of *G. arboreum*, *G. hirsutum*, *B. vulgaris*, and *Arabidopsis thaliana* to define genes that are well-annotated. This resulted in the identification of a total of 28 022 orthogroups (OGs) with genes of one or more species and only 6370 OGs were found to include genes from all five species. The groups containing DEGs in response to pH treatment are termed as DEG orthogroups, and gene expression data for them are shown in Supplementary Dataset S1. Most of the DEG orthogroups showed a different expression pattern in each species. Each DEG orthogroup might be comprised of a number of gene isoforms that differ by species; this could be due to the phylogenetic relationships between the species, and the number of gene isoforms belonging to a gene family can also vary by species. DEG orthogroups related to hormone response, calcium sensing, and photosynthesis were found to be differentially expressed in all species.

### Functional analysis

**Fig. 3.**
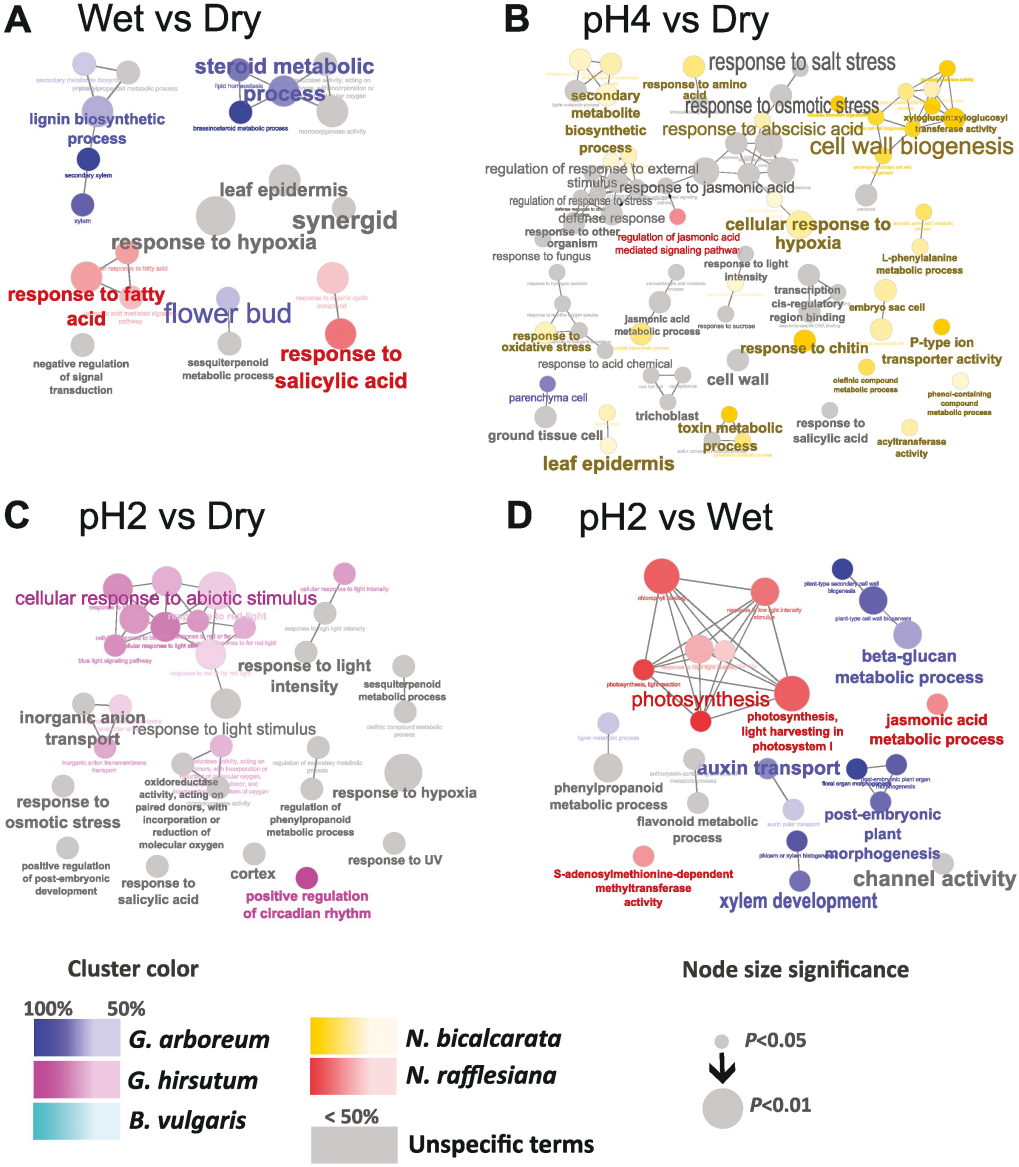
General plant responses to abiotic stresses are activated by wetting of the leaf surface and by changes in external pH. The pH of the leaf surface of *Gossypium arboreum*, *G. hirsutum*, *Beta vulgaris*, *Nepenthes bicalcarata*, and *N. rafflesiana* was determined (Dry control), and then they were sprayed with HCl solutions of pH 6.5 (Wet control), pH 4, and pH 2, and the resultant pH of the phylloplane was determined after 5 min. GO term enrichment of differentially expressed genes (DEGs) for the comparisons of (A) Wet versus Dry control, (B) pH 4 versus Dry control, (C) pH 2 versus Dry control, and (D) pH 2 versus Wet control. The circles indicate GO terms that are enriched with more than 50% of the DEGs from an individual species, as indicated in the key; grey circles are terms shared across the different species. The size of the circles represents the level of significance, as indicated in the key.

To determine which key plant metabolic pathways were involved in sensing or responding to wetness and pH changes, we performed a GO term enrichment analysis (Fig. 3). We found no metabolic categories enriched in the DEGs in the pH4vsWet comparison. The term ‘response to hypoxia’ was shared across species and shared in all three comparisons against the dry control (Fig. 3A–C). We observed unique responses in every comparison shared across species (gray circles in Fig. 3). The pH4vsDry comparison (Fig. 3B) resulted in the enrichment of the most terms related to signaling pathways in response to various abiotic stresses, such as salt stress, osmotic stress, oxidative stress, and to ‘external stimulus’, and also to biotic stresses including ‘other organism’ and ‘fungus’. In the pH2vsDry comparison (Fig. 3C), the ‘cellular response to abiotic stimulus’ was driven by *G. hirsutum*, but we still observed responses to ‘osmotic stress’ and to ‘light stimulus’ that were shared across the species. In the pH2vsWet (pH 6.5) comparison we could separate the response of the leaf to pH changes, excluding the response to wetness (Fig. 3D). Auxin transport and other metabolic processes involved in growth and development were affected by pH stress together with photosynthesis-related pathways. It could be observed that *G. arboreum* and *N. bicalcarata* were driving some of the GO-term categories in the WetvsDry and pH2vsWet comparisons, while the pH4vsDry comparison was enriched mainly by DEGs from *N. bicalcarata* (yellow circles in Fig. 3), and the pH2vsDry comparison by DEGs from *G. hirsutum* (pink circles).

**Fig. 4.**
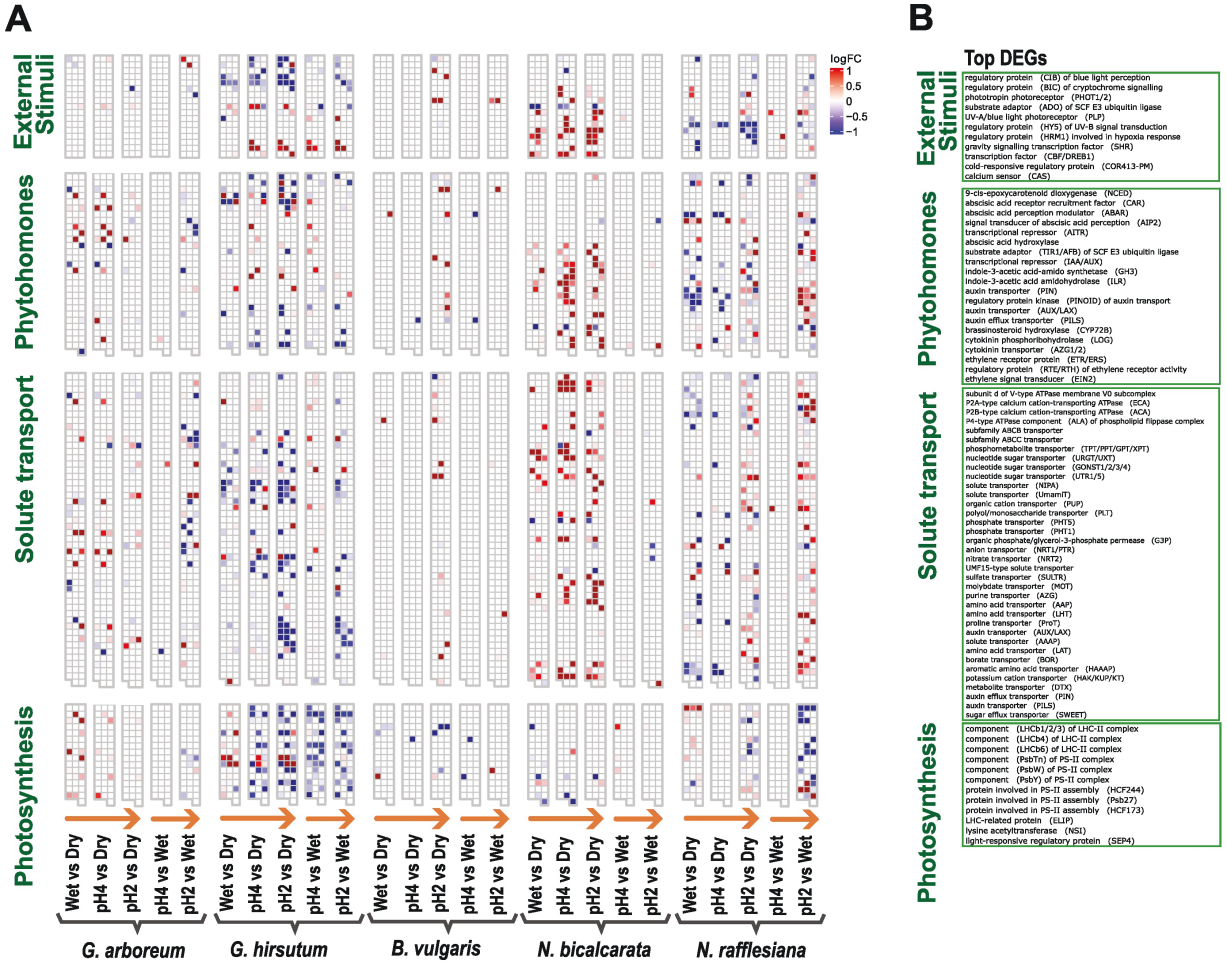
Gene expression patterns in response to changes in external pH on the leaf surface. The pH of the leaf surface of *Gossypium arboreum*, *G. hirsutum*, *Beta vulgaris*, *Nepenthes bicalcarata*, and *N. rafflesiana* was determined (Dry control), and then they were sprayed with HCl solutions of pH 6.5 (Wet control), pH 4, and pH 2, and the resultant pH of the phylloplane was determined after 5 min. (A) Heatmaps showing the expression patterns of orthologous DEGs across the species and treatment comparisons for selected metabolic categories of interest. The orange arrows represent increases in the acidification response. (B) List of the top 20 most highly expressed genes in each category.

We visualized the DEG orthogroups with Mapman to give a general overview of gene expression patterns that might be related to the pH gradient (Supplementary Dataset S1; Supplementary Video S1; Fig. 4). For example, Supplementary Fig. 1B shows that in *G. hirsutum* genes such as Light-regulated protein 1 (LIR1) and PSII repair protein PSB27-H1 were up-regulated in the WetvsDry and pH2vsDry comparison; however, following the pH gradient from neutral to more acidic indicates that there were many others that were down-regulated with decreasing pH, such as Light harvesting II (LHC-II) complex. Genes related to light reactions were expected to be differentially expressed due to pH changes in the chloroplast thylakoids/stroma being driven by light stimulus and the electron chain machinery, which helps to prevent photodamage. In *N. bicalcarata*, photorespiration was the main metabolic system where we observed up-regulation in the comparison against the dry control but not against the wet control (Supplementary Fig. S1D; Supplementary Video S1), which we can relate to wetness more than to pH changes, due to the pH treatment not causing a strong response in either of the *Nepenthes* species (Fig. 1A).

From this overview, we selected only the main metabolic categories that showed interesting expression patterns, namely external stimuli, phytohormones, solute transport, and photosynthesis (Fig. 4; Supplementary Dataset S1). As noted above, there were not many DEGs shared across species, but instead we observed that *G. hirsutum* and both the *Nepenthes* species each had more DEGs in each metabolism category (Fig. 4A). *Gossypium hirsutum* showed a pattern of increasing down-regulation of DEGs with decreasing pH, particularly in solute transport and photosynthesis, with the latter involving many genes related to the photosynthetic machinery such as the light harvesting complex (LHC) and PSII assembly. In contrast, *N. bicalcarata* had more up-regulation of DEGS, which was mainly observed in the treatment comparison against the dry control. In *N. rafflesiana*, more down-regulation of DEGs related to photosynthesis and more up-regulation of DEGs related to solute transport and phytohormones was observed in relation to the pH gradient.

### Co-expressed orthogroups across species

**Fig. 5.**
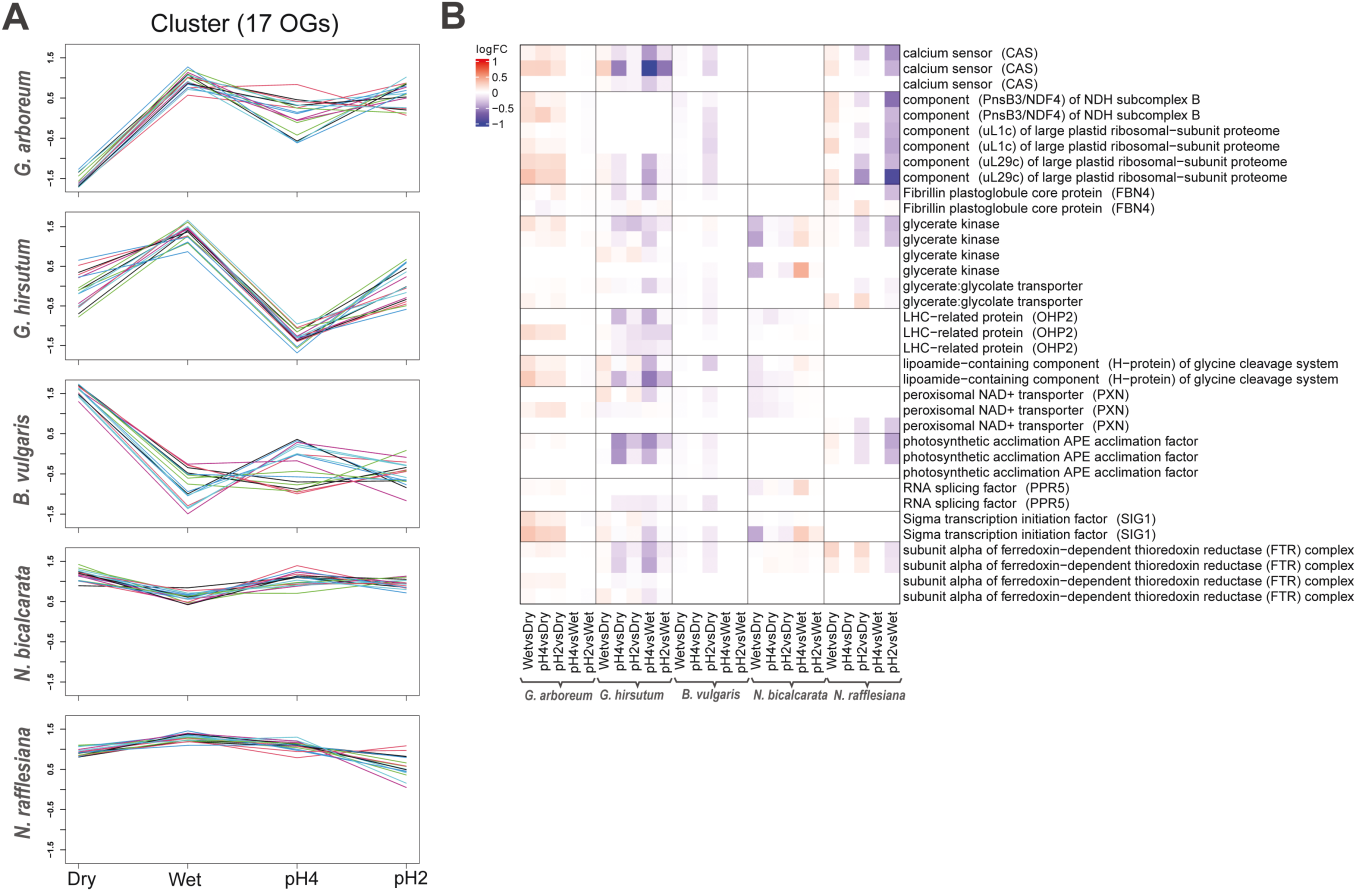
Genes related to the light-harvesting complex (LHC) and calcium sensors (CAS) are co-expressed across the species in response to changes in external pH on the leaf surface. The pH of the leaf surface of *Gossypium arboreum*, *G. hirsutum*, *Beta vulgaris*, *Nepenthes bicalcarata*, and *N. rafflesiana* was determined (Dry control), and then they were sprayed with HCl solutions of pH 6.5 (Wet control), pH 4, and pH 2, and the resultant pH of the phylloplane was determined after 5 min. The orthogroups matrix from OrthoFinder and subsequent cluster analysis with Clust identified only one cluster of 17 co-expressed orthogroups. (A) Overview of the orthogroup expression patterns across the different species and pH treatments. (B) Heatmap of the genes represented in (A).

To determine whether there were clusters of co-expressed genes shared across the species that might be involved in the response to pH changes, we used the orthogroups matrix from OrthoFinder and ran a cluster analysis using Clust. This identified only one cluster of 17 co-expressed orthogroups across the species. Whilst no clear pattern of gene expression could be seen in this cluster for *B. vulgaris*, a possible pattern driven by pH could be observed in the two *Gossypium* species, and a weaker effect could be seen in the two *Nepenthes* species (Fig. 5A). There were genes in this cluster related to the photosynthetic machinery (LHC) and to calcium sensors (Fig. 5B). More up-regulation within this cluster could be observed in *G. arboreum*, whilst more down-regulation was observed in *G. hirsutum*. No clear patterns were observed in *B. vulgaris* or either of the *Nepenthes* species.

## Discussion

Despite the likely importance of phylloplane pH in the context of abiotic (e.g. acid rain) and biotic (e.g. herbivores, microbes) stressors, it is a relatively understudied trait, with little known about the molecular mechanisms that plants might generally use to sense and regulate external pH homeostasis (Shepherd and Wagner, 2007; Gilbert and Renner, 2021). Here, we used a transcriptomic approach to examine differential gene expression in five plant species in response to three different acidic spray treatments on the phylloplane. Our results provide new insights at the molecular level that build upon previous knowledge gained from simulated acid rain studies by adding to the phylogenetic and physiological diversity of the pH-response data. Whereas such studies have usually focused on one or two species, typically selected solely for their agricultural relevance or status as model organisms, we selected a broader range of species spanning a range of baseline phylloplane pH extremes from highly alkaline to highly acidic. In addition, our study is novel in examining very short-term responses to acute pH changes, after 5 min of exposure to a single discrete pH stimulus, whereas past studies have utilized longer-term exposures of continuous acidic mist or fog treatments over periods of hours or days (reviewed by Gilbert and Renner, 2021). Furthermore, our experiment was designed to examine the external pH effects per se, not those of (chemically complex) acid rain, and hence our use of HCl as a simple source of H^+^ ions to produce a gradient of different pH treatments. It was not our aim to determine a definitive mechanism of phylloplane pH regulation, as that would require more precise experiments such as gene knockdown; however, our approach has revealed several interesting patterns that can inform future studies. One major finding was the unexpected differences in molecular responses across species, even within the same genus; however, we also found some shared patterns across species, pointing to the potential involvement of calcium ions in the response to pH and to the general negative impact of pH stress on photosynthesis. In addition, we consider the potential integration of plant pH molecular response into a core abiotic/biotic stress signaling pathway.

### Phylloplane buffering ability is species-specific

We observed that external pH changes had a variable impact on the phylloplane of different plants, which was reflected in the variation of each species’ molecular response (Figs 1, 4). In the comparative transcriptomic analysis, the species showed different numbers of DEGs across the different pH treatment comparisons (Fig. 2). A similar pattern of interspecific variation was found in a previous comparative transcriptomic analysis across Arabidopsis, *Hordeum vulgare*, and *Oryza sativa* in response to different abiotic stresses, including responses of hormones such as abscisic acid and salicylic acid (Hartmann *et al.*, 2022). Our expectation that a conserved molecular mechanism underlies responses to external pH disturbances is contradicted by these findings. Species-specific molecular responses might indicate that other factors influence how plants sense external environmental changes, including novel molecular mechanisms, differences in morphological or anatomical traits, and/or phylogenetic relationships.

One relevant phenotypic characteristic that is likely to be of importance is differences in leaf morphological protective mechanisms (e.g. trichomes and waxy cuticles). Cuticles play an important role in transpiration, the loss and uptake of polar solutes, and biotic interactions (Hauser *et al.*, 1993; Zeisler-Diehl *et al.*, 2018). Harr and Guggenheim (1995) described properties of leaf surfaces in different crop species, including the contact angle of water on the leaf surface (e.g., 34° for *G. hirsutum*, 65° for *B. vulgaris*) and the chemical composition of cuticular waxes, highlighting the differences in the chemistry of the cuticles between *B. vulgaris* and *G. hirsutum*. The composition of waxy cuticles can make leaf surfaces impermeable to water and dissolved solutes, and control how they interact with microorganisms including potential pathogens (Zeisler-Diehl *et al.*, 2018; Wang *et al.*, 2020). Alterations of waxy cuticular composition have been observed under extreme acid rain (pH 2.5 Rodríguez-Sánchez *et al.*, 2020); however, this can be species-dependent and influenced by the acidity level, as well as the frequency and exposure time of the acid rain event (Harr and Guggenheim, 1995; Shi *et al.*, 2021). As such leaf-surface properties are fixed developmental differences, the different pH responses among *B. vulgaris* and *Gossypium* species are not necessarily only due to active response mechanisms. Future studies of variation in cuticular composition could help to better explain how passive and active mechanisms counteract environmental changes.

The observed differences in response to pH disturbance (Fig. 1) might be related to phylogenetic differences as well. *Beta* and *Nepenthes* are members of *Caryophyllales* (Gilbert and Renner, 2021), whilst *Gossypium* is within *Malvales*. *Gossypium* species are known to have an alkaline pH on their leaf surfaces (Harr *et al.*, 1980), while species in *Nepenthes* tend to have broad range of acidic pH (pH 1–5; Takeuchi *et al.*, 2015; Gilbert and Renner, 2021). We found that *B. vulgaris* had the lowest number of DEGs resulting from our pH treatments (Fig. 2). The response of *B. vulgaris* to such pH changes can probably be extrapolated to most species, given that its phylloplane pH was what we would consider as being ‘physiologically typical’. However, additional research on other possible passive mechanisms is necessary. In our differential gene expression analysis, we found that not only did the composition of DEGs differ among the species, but they were linked to functional differences as well. In this regard, we expected the pairs of *Gossypium* and *Nepenthes* species to share genus-level similarities among the expressed metabolic pathways, and yet we found dissimilarities within the genera. Although both *Gossypium* species showed similar buffering of pH (Fig. 1), and had a similar number of total DEGs, the latter varied across the treatment comparisons (Fig. 2). This mismatch in expression patterning was also observed in the two *Nepenthes* species, again highlighting how the molecular mechanisms sensing and responding to external pH disturbances can vary with species. Overall, many of the GO terms were driven by individual species, with ‘secondary xylem’ in ‘lignin biosynthetic process’ and ‘brassinosteroids metabolic process’ being enriched in *G. hirsutum* while ‘response to fatty acid’ and ‘response to salicylic acid’ were enriched in *N. bicalcarata* (Fig. 3A). In addition, we were expecting a similar sensing response of the Cl^–^ ion from the HCl treatment in each species, at least for the pH 2 treatment where an impact on the buffering ability was observed for all the species. Surprisingly, however, only *B. vulgaris* and *N. rafflesiana* showed up-regulation of the *CLC* chlorine sensor gene in the pH2vsWet comparison (Supplementary Dataset S1), which might suggest that these two species were relatively more sensitive to Cl^–^.

The two *Nepenthes* species showed the highest numbers of DEGs, suggesting that they possess broader molecular mechanisms to detect their surrounding environment (Figs 2, 4). It is worth noting that *N. gracilis* was recently determined to be decaploid (Saul *et al.*, 2023). Unlike the other species in this study, the *Nepenthes* were subjected to a constantly high-humidity environment (80%), reflecting their native tropical habitat. More moisture can lead to an increase in biotic interactions with microbes and insects (Evans *et al.*, 2022; Lebrija-Trejos *et al.*, 2023), which could mean plants need to develop more complex/reactive mechanisms for defense and sensing their surroundings. For instance, one of the most highly enriched GO terms for *N. bicalcarata* was ‘response to other organism’ and for *N. rafflesiana* ‘regulation of defense response’ (Supplementary Fig. S1), and other studies have shown how pathogens can activate plant defense mechanisms through changes in extra- and intracellular pH (Li *et al.*, 2025). Further studies are needed to characterize deeper anatomical and chemical properties of leaves that might explain why, for some plants, it can be more beneficial to buffer external pH changes, whilst for others their physical/chemical leaf properties might be enough to cope with external disturbance.

### Ca^2+^ plays a role in the response to pH stress

The regulation of phylloplane pH is underlain by control of the flux of ions into and out of cells, and we expected that pathways related to active transport of ions would be involved in the plant response to pH. Indeed, Ca^2+^ was revealed to be a key part of the phylloplane response to external pH across all the species in our study. To our knowledge, the relationship of Ca^2+^ and pH homeostasis in this context has not been widely addressed previously, although a recent study found that acid stimulus can influence intracellular calcium levels (Chen *et al.*, 2024). Studies on drought and salt stress have shown a relationship between pH and Ca^2+^: when levels of Ca^2+^ increase in the cytosol, there is a concomitant decrease in pH (Gao *et al.*, 2004; Feng *et al.*, 2016; Ruan *et al.*, 2024). Other studies have reported a similar relationship between calcium and pH changes in the cytosol in response to wounding, extracellular ATP, and treatment with a synthetic auxin (Behera *et al.*, 2018). In our study, calcium-related genes were found to be differentially expressed in response to pH, such as the vacuolar cation/proton exchanger *CAX*, which was down-regulated in all the treatment comparisons in *G. hirsutum*, and calcium sensors that were found in the conserved cluster across all the species (Fig. 4; Supplementary Dataset S1). CAX antiporters are involved in the transport of Ca^2+^ and other cations using the H^+^ or Na^+^ gradient generated by primary transporters (Shigaki *et al.*, 2006; Pittman and Hirschi, 2024). In addition to the presence of intracellular Ca^2+^, these ions might also be transported to the external environment on the phylloplane, as seen in *G. hirsutum* (Elleman and Entwistle, 1982). Salt-excreting glands or hydathodes in cuticles are known to modulate the loss or uptake of solutes (Elleman and Entwistle, 1982; Zeisler-Diehl *et al.*, 2018), and the transport of a wide range of solutes is one of the basic metabolic processes involved in how plants sense their external surroundings. According to the metabolic categories identified by our Mapman analysis, solute transport seemed to be highly active and differentially regulated across the pH gradient that we studied, particularly in *G. hirsutum*, *N. bicalcarata*, and *N. rafflesiana* (Fig. 4).

Transporters of inorganic elements (Ca^2+^, K^+^, and P), organic compounds (sugars and amino acids), and hormones (auxin) were found to be differentially expressed (Fig. 4B; Supplementary Dataset S1). Although a high-affinity K^+^ transporter (HAK) gene was down-regulated in the pH2vsWet comparison in *G. arboreum* (*HAK2*) and in the pH4vsDry comparison in *N. rafflesiana* (*HAK5*), *HAK19* was up-regulated in the pH4vsDry and pH2vsDry comparisons in *N. bicalcarata*. HAK transporters are part of the H^+^/K^+^ symporter family, and they have been found in the tonoplast or plasmalemma in the leaves of different species including rice and barley; they are involved in multiple processes related to K^+^ uptake, cell osmoregulation, and turgor maintenance (Shabala, 2003). The role of K^+^ in *Nepenthes* might be related to the active solute transport mechanism that is triggered by wetness and pH changes. Cations including Ca^2+^, Mg^2+^, and K^+^ have been found to accumulate on the leaf surfaces of multiple species of *Gossypium* (Harr *et al.*, 1980), and Elleman and Entwistle (1982) determined that glandular trichomes in *G. hirsutum* might be responsible for excretion of crystals of these ions to the surface and the concomitant increased alkalinity that is characteristic of this genus and the *Malvales*. Thus, the differential expression of the Ca^2+^ signaling pathway in response to the application of external acidic stimuli seems suggestive of some form of physiologically active transport connected to the observed buffering phenotype, which was strongest for the *Gossypium* species (Fig. 1). On the other hand, the Ca^2+^-signaling pathway is generally the major point of signaling crosstalk between abiotic and biotic stresses (Dong *et al.*, 2022; Li *et al.*, 2022; Xu *et al.*, 2022). An active metabolism involving Ca^2+^-ATPases and calcium sensors seemed to be responsive in most of our species (Figs 4B, 5B).

Regarding our observations for Ca^2+^-ATPases, we were also expecting to find H^+^-ATPases that were differentially expressed in response to external pH changes. We searched for plasma membrane ATPases in *Gossypium* due to the stronger buffering ability in this genus, basing our search on the H^+^-ATPase annotation reported by Chen *et al.* (2018). We were able to identify a V-type H^+^-ATPase pump among our DEGs, which was up-regulated in the pH4vsWet comparison in *G. hirsutum* (Supplementary Dataset S1). V-type proton ATPases are primarily involved in intracellular pH regulation by pumping H^+^ out of the vacuolar membranes (Müller *et al.*, 1996; Liang *et al.*, 2015). This finding also highlights the role of the vacuoles in the regulation of pH: they are known to store different types of acid compounds to maintain the homeostasis of intracellular pH, as well as playing a critical role in signaling pathways for abiotic and biotic stress (Faraco *et al.*, 2014; Zhang *et al.*, 2014; Martinoia *et al.*, 2018). We did not find differentially expressed H^+^-ATPases in *G. arboreum,* but this might be related to genetic differences between the two *Gossypium* species. Notably, *G. arboreum* is a diploid (AA genome), while *G. hirsutum* is an allotetraploid (AD genome; Li *et al.*, 2014, 2015). In addition, the two species have a variable number of genes belonging to the H^+^-ATPase family (Chen *et al.*, 2018). The role of proton-pump ATPases in our study was not completely clear, and was possibly masked by other unexamined factors, such as morphological features and other consequences of evolutionary history.

### Photosynthesis is negatively affected by pH stress

Photosynthesis is one of the main metabolic pathways to come to mind when considering leaves. Studies on acid rain have shown the negative effects of the stress particularly in the photosynthetic machinery (Sun *et al.*, 2016; Shi *et al.*, 2021). We found that genes involved in photosynthetic machinery and responses to light seemed to be differentially expressed in response to pH and were mostly down-regulated following the increase in acidity. This was most apparent in *G. hirsutum* and *N. rafflesiana*, and in the pH2vsWet comparison seen in the GO term enrichment (Figs 3D, 4). Shi *et al.* (2021) reported inhibition of photosynthetic rates under acid rain with pH ranging from 2 to 4. Genes related to photosynthesis were found to be differentially expressed across species, and it was clearly affected by pH stress, mainly in *G. hirsutum* and *N. rafflesiana* (Fig. 3D; Supplementary Fig. S1; Supplementary Dataset S1). The stroma, in which the photosynthetic machinery is located, is known to be susceptible to changes in intracellular pH (Aranda Sicilia *et al.*, 2021), with acidification generally leading to disruption involving the photosystem and the electron transport chain, which is followed by breakage of chlorophyll (Tikhonov, 2012; Trinh and Masuda, 2022). Genes encoding the LHC and PSII in particular were down-regulated in all the species (Fig. 4; Supplementary Dataset S1), probably inhibited due to acidification of the stroma (Trinh and Masuda, 2022).

Our results are in agreement with the negative effects of acidic pH stress on the photosynthetic machinery that have been reported before with simulated acid rain experiments (Sun *et al.*, 2016; Shi *et al.*, 2021), and they also highlight other possible previously unidentified active and/or passive mechanisms such as morphological/anatomical properties that can lead to differences in the response to the same pH treatment, as seen across the species in our study. The negative impact of pH stress on photosynthesis can have repercussions from an ecological point of view in terms of the effects of increases in external acidification agents such as acid rain, foliar pesticides, and fertilizers. Hence, it might be beneficial to study how external acidification can affect crops of commercial interest.

### The molecular response to pH is interlinked with other abiotic and biotic stress-signaling pathways

In nature, plants commonly interact with combinations of stressors at any given time, and the response to such multiple stresses can be interconnected in a major signaling pathway to accommodate faster and more efficient responses in a changing environment (Zandalinas *et al.*, 2020). Responses to different types of abiotic and biotic stresses have been observed to converge within the molecular pathways (Pereira, 2016; Zandalinas *et al.*, 2020; Li *et al.*, 2022; Munns and Millar, 2023; Zhang *et al.*, 2023). Here, we found that DEGs related to different abiotic and biotic responses were mostly activated in the pH 4 treatment, and the *Nepenthes* species were the major contributors to the DEGs found in this shared pathway (Fig. 3). This suggests that leaves sensing external mild pH changes activate other abiotic stresses, or the general response to abiotic stress in which pH is integrated as a stressor. One of the few shared GO terms enriched across all the species in all the comparisons with the dry control was related to ‘hypoxia’ (Fig. 3A–C), suggesting that they were all sensing wetness (i.e. a slight reduction in oxygen on the surface when covered in water droplets). Sensing wetness on the leaf surface seemed to activate biotic responses, as we can infer from the GO terms ‘interaction with other organism’ and ‘response to fungus’ (Fig. 3B). Plant responses to biotic stress can trigger the production of salicylic acid, jasmonic acid, and ethylene depending on the pathogen (Cohen and Leach, 2019), and related GO terms were also enriched in our results. Understanding the ecological implications of this buffering ability in the interactions with other organisms such as microbes and insects seems to be a worthwhile topic of research.

Plant responses to abiotic stresses such as drought, cold, salinity, and wounding share a core signaling pathway (Verma *et al.*, 2016; Kidokoro *et al.*, 2017; Skalak *et al.*, 2021). Perception of these stresses starts with membrane receptors, such as G protein-coupled receptors, receptor-like kinases, histidine kinases, and ion channels. These receptors lead to changes in the pool of Ca^2+^, which activate several kinases, such as calcium-dependent protein kinases (Xu *et al.*, 2022). However, even when the plant uses similar players for different stresses, the final section of the response mechanism is more specific to the type of stress (Cramer *et al.*, 2011).

## Conclusions

Our comparative transcriptomic analysis of leaf responses to external pH changes showed how the effect of the stress differed between species. The variations we observed in the molecular response to the pH changes help to expand our knowledge of how plants sense their surrounding environment in a unique way that can be related to their specific traits, such as morphological/anatomical characteristics, phylogenetic relationships, and physiological factors. This has relevance in the context of the increasing acidity in the environment due to pollution and/or the use of foliar pesticides/fertilizers and how each species might be affected and respond in a different way. We found that a mild pH change triggered the activation of signaling-pathway genes that are involved in responses to many other abiotic stresses, such as osmotic and drought stress. Furthermore, we observed pathways related to interactions with other organisms and defense pathways were turned on in response to wetness and mild pH. *Gossypium* was the genus that was best able to buffer against acidic pH, and its response incorporated the participation of Ca^2+^-related metabolism, which might be involved in its alkalinizing phenotype, although further studies are needed to test its role in the regulation of external pH. We observed that differentially expressed genes varied across the species and that might have been associated with the variation of the effects of pH, but the differences in the molecular responses were more complex. For example, we expected to find H^+^-ATPases involved as part of a general mechanism in regulating phylloplane pH, but interestingly we only found such a vacuolar proton pump to be differentially expressed in *G. hirsutum*. The lack of similar responses between closely related species was surprising, particularly for the two *Gossypium* species where the observed phylloplane pH phenotypes were quite similar. Further studies are needed in other species of *Gossypium* that vary in their buffering ability in order to characterize the enzymes that we have identified in this study to further clarify the underlying molecular mechanism(s). Further work is also needed to determine how the pH responses can be integrated into a core abiotic stress pathway. On the basis of our results, we can conclude that the impact of external pH stress is species-specific, and it is important to achieve a deeper understanding of how the transcriptional responses are linked to other protective mechanisms within the plant. This could also help to estimate the ecological impact of the increasing acidification of the environment due to pollution.

## Supplementary data

The following supplementary data are available at *JXB* online.

Fig. S1. Functional analysis of DEGs by species.

Table S1. Results of generalized linear model analysis of the resultant pH for the different species and treatments, and their interactions.

Dataset S1. Differentially expressed genes by treatment comparison across the species with annotations and identities of orthogroups.

Video S1. Visualization of changes in gene expression by treatment across the species.

eraf157_suppl_Supplementary_Tables_S1

eraf157_suppl_Supplementary_Figure_S1

eraf157_suppl_Supplementary_Datasets_S1

eraf157_suppl_Supplementary_Videos_S1

## Data Availability

The raw RNA-seq data have been deposited in the NCBI Gene Expression Omnibus database (https://www.ncbi.nlm.nih.gov/geo/) under accession number GSE281272.
