# Supplementary material for: Species-specific phylloplane responses to changes in external pH
Source: J Exp Bot. 2025 Apr 14;76(17):5102–16. doi: 10.1093/jxb/eraf157 (PMC12587422; doi:10.1093/jxb/eraf157)
Supplement: eraf157_suppl_Supplementary_Tables_S1 [file eraf157_suppl_supplementary_tables_s1.pdf]

Supplementary Table S1. Resultant pH was significant different in each species compare to beta vulgaris. The pH2 treatment significantly changed the resultant pH in each species whereas only pH4 have a significant impact on G. arboreum. Generalized linear model (gml) of resultant pH versus species and treatment and their interactions; p-value, '***' 0.001, '**' 0.01, '*' 0.05.

Pairwise comparison	Coefficient of variation	Standard Error	p-value
(Intercept)	7.16	0.3062	< 2.00E-16 ***
SpeciesGossypium_arboreum	1.5733	0.4331	7.89E-04 ***
SpeciesGossypium_hirsutum	1.8633	0.4331	1.06E-04 ***
SpeciesNepenthes_bicalcarata	-2.2933	0.4331	4.60E-06 ***
SpeciesNepenthes_rafflesiana	-1.6533	0.4331	4.59E-04 ***
TreatmentpH2	-4.63	0.4331	2.72E-13 ***
TreatmentpH4	-0.1967	0.4331	0.65
TreatmentWet	0.2367	0.4331	0.59
SpeciesGossypium_arboreum:TreatmentpH2	1.71	0.6125	8.00E-03 **
SpeciesGossypium_hirsutum:TreatmentpH2	1.4467	0.6125	0.02 *
SpeciesNepenthes_bicalcarata:TreatmentpH2	2.2133	0.6125	8.34E-04 ***
SpeciesNepenthes_rafflesiana:TreatmentpH2	1.5267	0.6125	0.02 *
SpeciesGossypium_arboreum:TreatmentpH4	1.55	0.6125	0.02 *
SpeciesGossypium_hirsutum:TreatmentpH4	0.9	0.6125	0.15
SpeciesNepenthes_bicalcarata:TreatmentpH4	-1.05	0.6125	0.09
SpeciesNepenthes_rafflesiana:TreatmentpH4	-0.88	0.6125	0.16
SpeciesGossypium_arboreum:TreatmentWet	1.1933	0.6125	0.06
SpeciesGossypium_hirsutum:TreatmentWet	0.7467	0.6125	0.23
SpeciesNepenthes_bicalcarata:TreatmentWet	-1.1433	0.6125	0.07
SpeciesNepenthes_rafflesiana:TreatmentWet	-0.8967	0.6125	0.15
