# Supplementary material for: Species-specific phylloplane responses to changes in external pH
Source: J Exp Bot. 2025 Apr 14;76(17):5102–16. doi: 10.1093/jxb/eraf157 (PMC12587422; doi:10.1093/jxb/eraf157)
Supplement: eraf157_suppl_Supplementary_Figure_S1 [file eraf157_suppl_supplementary_figure_s1.pdf]

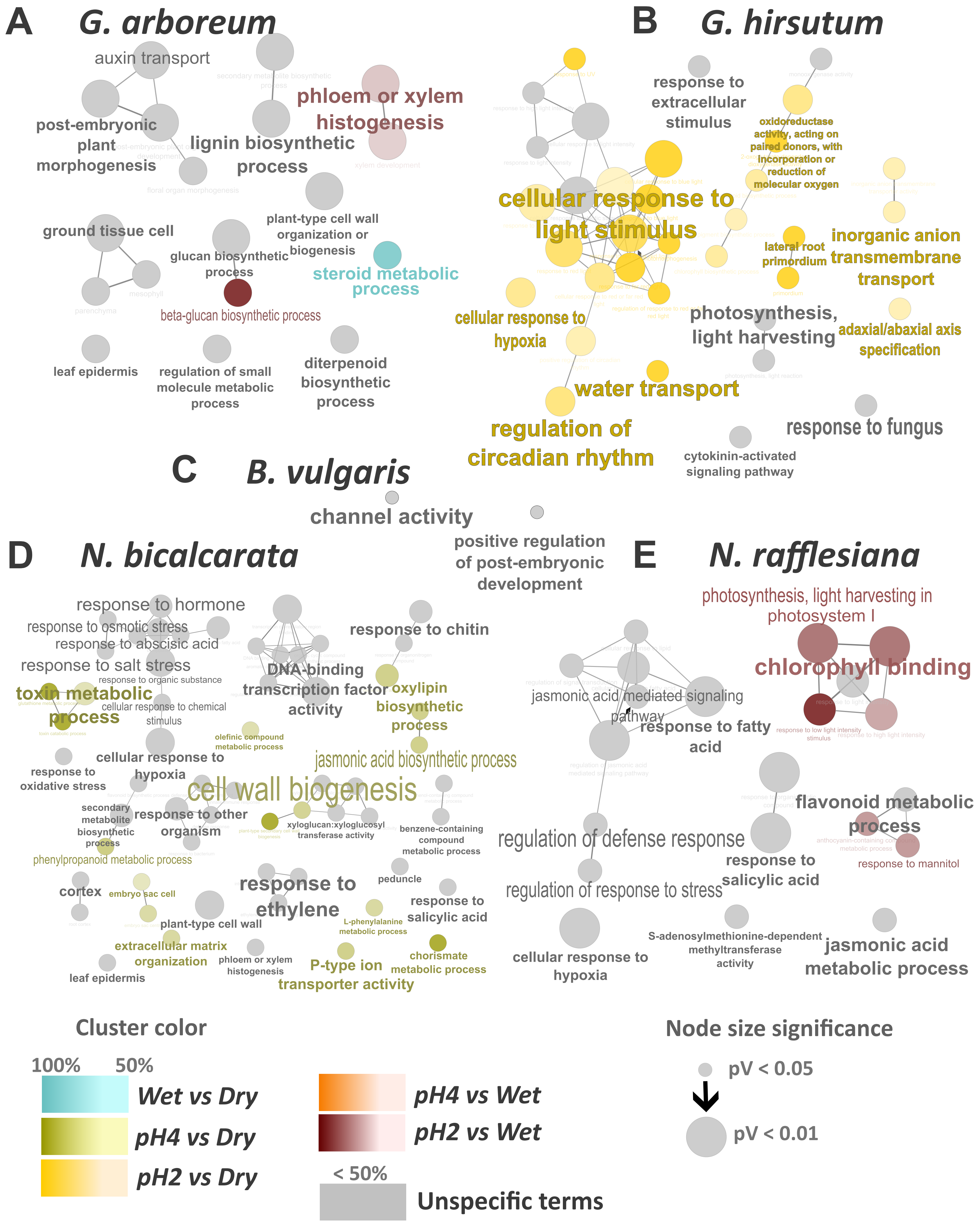

Supplementary Figure S1. Most of the variation on the functional response to pH treatment is driven by species. A) to E) are the GO term enrichment of DEGs by species. Grey circles are GO terms shared across different DEGs treatment comparison. Circles with different colors are GO terms that are enriched with more than 50% of the DEGs from an specific treatment comparison described in the legend. The size of the circles represent how significant they are, the smallest started from p-value of 0.05. "Response to salicylic acid" is a GO term shared across treatment comparison in both *Nepenthes* species.
