# Supplementary figures and images for: Species-specific phylloplane responses to changes in external pH
Source: J Exp Bot. 2025 Apr 14;76(17):5102–16. doi: 10.1093/jxb/eraf157 (PMC12587422; doi:10.1093/jxb/eraf157)

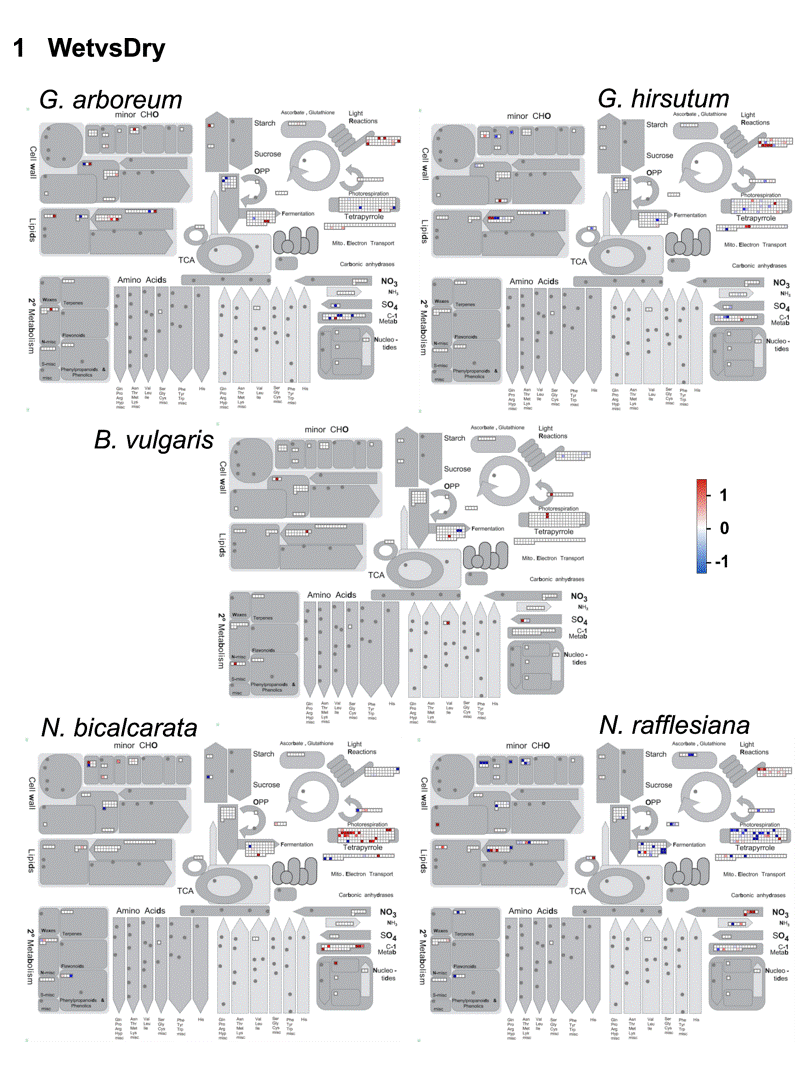

Supplement: eraf157_suppl_Supplementary_Videos_S1 [file eraf157_suppl_supplementary_videos_s1.zip › eraf157_suppl_Supplementary_Videos_S1.gif]
